# Computational ensemble expert system classification for the recognition of bruxism using physiological signals

**DOI:** 10.1016/j.heliyon.2024.e25958

**Published:** 2024-02-10

**Authors:** Pragati Tripathi, M.A. Ansari, Tapan Kumar Gandhi, Faisal Albalwy, Rajat Mehrotra, Deepak Mishra

**Affiliations:** a Department of Electrical Engineering, Gautam Buddha University, Greater Noida, India; b Department of Electrical Engineering, Indian Institute of Technology Delhi, India; c Department of Computer Science, College of Computer Science and Engineering, Taibah University, Madinah, Saudi Arabia; d Division of Informatics, Imaging and Data Sciences, Stopford Building, University of Manchester, Oxford Road, Manchester, M13 9PL, UK; e Department of Examination & Analysis, Amity University, Noida, India; f Department of Computer Science, College of Vocational Studies, University of Delhi, India

**Keywords:** Ensemble machine learning classifier, EEG, ECG, EMG, Bruxism, Power spectral density (PSD)

## Abstract

This study aimed to develop an automatic diagnostic scheme for bruxism, a sleep-related disorder characterized by teeth grinding and clenching. The aim was to improve on existing methods, which have been proven to be inefficient and challenging. We utilized a novel hybrid machine learning classifier, facilitated by the Weka tool, to diagnose bruxism from biological signals. The study processed and examined these biological signals by calculating the power spectral density. Data were categorized into normal or bruxism categories based on the EEG channel (C4-A1), and the sleeping phases were classified into wake (w) and rapid eye movement (REM) stages using the ECG channel (ECG1-ECG2). The classification resulted in a maximum specificity of 93% and an accuracy of 95% for the EEG-based diagnosis. The ECG-based classification yielded a supreme specificity of 87% and an accuracy of 96%. Furthermore, combining these phases using the EMG channel (EMG1-EMG2) achieved the highest specificity of 95% and accuracy of 98%. The ensemble Weka tool combined all three physiological signals EMG, ECG, and EEG, to classify the sleep stages and subjects. This integration increased the specificity and accuracy to 97% and 99%, respectively. This indicates that a more precise bruxism diagnosis can be obtained by including all three biological signals. The proposed method significantly improves bruxism diagnosis accuracy, potentially enhancing automatic home monitoring systems for this disorder. Future studies may expand this work by applying it to patients for practical use.

## 1 Introduction

Sleep is a common and crucial requirement for all living beings. It is defined as the alteration in consciousness, restriction in sensory movements, and reduced muscle movements [1]. It is also found in all living beings, such as reptiles, mammals, Pisces, animals and humans. Some animals sleep with one eye open, and some sleep with both eyes closed. Sleep is categorized into four categories, including REM, NREM, wake and sleep. Lack of sleep influences the person's health, leading to various mental disorders like mood swings, memory loss, lack of concentration, heart disorders, enhanced risk of stroke and brain hemorrhages, obesity, and diabetes. Lack of sleep disastrously affects a person's ability to function, their health and the mental and emotional balance of the body. Good quality of sleep is a symbol of good health. It is generally said that reduced sleep can lead to decreased efficiency, reduced ability to handle stress and mood swings.

Continuously ignoring sleep can damage a person's health as much as caused by accidents, frustration, and lack of performance and according to AASM (American Academy of sleep medicine), USA and their physicians reported that 50–70 million people are affected by sleep disorders (https://www.cdc.gov/sleep/data_statistics.html). According to an Indian report, about 93 per cent of people are affected by sleep disorders [2]. About 70–75 per cent of people in the UK and European countries are affected by sleep disorders. Sleep disorders are mainly categorized as sleep disorders, sleep apnea, and unusual sleep patterns [3]. Bruxism is a kind of snooze disability in which the person unconsciously gathers, chew and gnash their teeth. Bruxism has been categorized into mild and severe types of sleep disorders [4].

The disturbance in sleep has many adverse effects in the long run like poor functioning in day-time like lack of concentration, poor efficiency, irritation, reduced reaction times. An investigation has been performed to give a brief on health effects like it affects metabolic impact, problem in nervous system, abnormal cardiac rhythm. In normal adults, it leads to very less period effects like stress, decreased quality of life, emotional breakdown and mood problems and disturbed nervous activity, memory and pursuance problem. For teenagers, the mental health gets affected also disturbs the pursuance in school and change in behaviors. The behavior issues are combined with the disturbance in sleep for children. Long term effects are that there is disturbance in sleep and healthy people get problem of diabetes mellitus, hypertension, cardiovascular problem, weight issues and cancer. Mostly mortality issues generate due to lack of sleep. In clinical situations, sleep disturbance can cause bad quality of life for children and teenagers and may cause gastric problems. Due to which, the work efficiency may affect and can cause the endurance in sleep and may assume interferences which can decrease sleep distortion [5].

Mild bruxism does not require treatment, but severe bruxism requires immediate treatment as it can damage teeth, cause headache, jaw problems and other mental disorders [6].

Bruxism is a paradigm that unconsciously affects the teeth grinding in a rhythm [7]. The combined effect can lead to the breaking and flattening of teeth, increased pain in teeth and enhanced sensitivity. Bruxism is divided into two categories, namely Sleep bruxism and wake bruxism. If the person grinds or chews the tooth at the time of wake-up, it is known as wake bruxism, and when the subject chews the tooth during sleep, that is known as snooze bruxism [8]. The indications of bruxism are jaw dysfunction, abnormal shape of teeth and jaw weakening, broken and clenching of teeth, sensitivity, severe headache and lack of sleep [9]. Sleep bruxism in the simple terms is a monotonous mechanical event with tooth crushing. Because of its medical performance and symptoms, the detection and organization of this situation is frequently followed by medical experts of sleep and dentists. Though, the descriptions and organization choices frequently distinct among the two areas, making misunderstanding and absence of clearness around sleep bruxism and creating the associations among them is most tedious. In sleep medicine, sleep bruxism was in beginning categorized as a parasomnia, but around 2005, according to the sleep doctors it is now assumed to be drive condition.

A general understanding of the ICSD is to assume all sleep bruxism cases as a syndrome. For a sleep motion nature to be categorized as a syndrome, the below requirements to be understand: Night time sleep trouble or problems of daytime sleep or dizziness are basics for the detection of a sleep linked motion illness [10]. Sleep bruxism includes the crushing of teeth at the time of sleep giving rise to distortion in teeth whereas RMMA (rhythmic masticatory muscle activity) includes the rhythmic event without clenching of teeth which is commonly observed in nervous situations. Both of them can affect quality of sleep and give rise to distortion of jaw but both of them have peculiar conditions and might need distinct techniques to adjust its cure. It is evident that ICSD i.e., International Classification of Sleep Disorders were formed for the clinical situation where sleep footages are mostly utilized for detection in complicated belongings as sleep bruxism is called to be combined with pains, sleep conscious problems, random eye movement (REM), sleep nature problems or sleep epilepsy [11]. Though, ICSD standards are often used in medical dentistry, where the footage is incomplete to single channel equipment's that have the main aim of printing or nursing cases.

The standards cannot just be written but validated in distinct age that is young age children, adults and old age people. In the dental area, sleep bruxism was assumed to be dysfunction an event happening in similar to masticatory activity. In a recent case published in 2013, advised that sleep and conscious muscle events in then normal persons, bruxism should not be assumed to be problem but rather than as a nature and bruxism related masticatory muscle events should be evaluated in the nature's continuation, dentists generally form sleep bruxism detection taking into consideration its existence during awakening, tooth problem and criticism of irritating noises [11]. Though, they also admit a probable collaboration to pain in head joint pain, snoring, and many more different neurological situations. Dentists have always been busy in the bad behavior of bruxism event and multiple problems creates when the overhead problems are detected: clinical detection and dental restoration plans [12]. In that side, a clinical appeal for detection and sleep footages is required. In lieu of the fact that the parts and characters of sleep bruxism distinct among doctors and dentists, clinical dental association is compulsory to get the optimal organization when sleep breathing situations are suspected in children, adults [13].

The detection of bruxism is accomplished in various ways, like investigating previous diseases, any other investigations, electric transplants in the mouth to see for the parameters, and teeth recording to see how intense the disease is [14]. Accurately detecting bruxism is a costly process that needs investigation in a proper sequence, more often in sleep medical spots, to estimate the various features and bodily symptoms during sleep [15]. Various signals like cardiac signal, EOG signal, which is related to the eye, EEG signal, which is related to the brain and EMG signal, which is related to muscle, are also utilized for detecting the type of physical signs at the time of sleep [16]. Guillot et al. [17] investigated the medical research of dentists belonging to France.

The novelists utilized 1388 medical practitioners, which detected the which is based on five approaches: mouth restoration, personal medical support with occlusal splints, which have social and demographic features, and detection and management of sleep bruxism. The users practiced almost 16.8% broad distinctiveness and the accessibility of satisfactory detection, with almost 21.9% of candidates proposing mental and emotional exercise [18,19]. Saczuk et al. [20] investigated an approach to detecting bruxism. The research scientists utilized sixty youths, involving 25 normal and 35 with bruxism disorder. They perform testing of the purpose of the physical muscles. Mada et al. [21] examined the validation of bruxism diagnosis of bruxism utilizing an electromyogram signal and the grade of interruption with more sensitivity and lucidity. We propose a new detection system for bruxism using the extraction of power spectral density on physiological sleep recordings such as ECG (ECG1-ECG2), EMG (EMG1-EMG2), and EEG (C4-P4 and C4-A1). Initially, the physiological signal is extracted from the sleep database. An entire 16 subjects have been considered in this manuscript.

The researchers have concluded that EMG for a unified channel and an optimum number are exactly for the diagnosis of bruxism disability. Mietinen et al. [22] advised that the ambulant electrodes are the most precise approach for detecting bruxism sleep disease and receiving themes on the stages of sleep. Ruhlund et al. [23] signify the detection of sleep bruxism by applying the human muscles through an EMG signal. Martines et al. [24] examined that the far networks and electric nerves would be utilized to detect bruxism. Kostku and Tkacz [25] explored the different data sources with stress imbalances applicable for the initial detection of phases of bruxism. Jirakittayakom and Wongsawat [26] found the EMG, a muscle signal used to detect sleep bruxism patients for the big muscles. In this manuscript, a novel scheme has been designed to detect bruxism by applying the most crucial parameter, spectral density energy, in the recordings of the sleep stages of the channels taken into consideration. Primarily the signal for life is applied from the area of sleep. A 10/20 snooze general footage scheme has been used for recording the dataset. This type of dataset is used for sleep disorders, circadian rhythm disturbances, insomnia [[27], [28], [29], [30], [31]], bruxism [[32], [33], [34]], sleep apnea, leg syndrome, narcolepsy [35], and previous epilepsy [36]. It is applicable in the analysis of sleep disabilities. Here the signals are taken by using (FIR) riddle to terminate the clatter of the signal by passing the filtering approach to the spectral density of the strong signal. In conclusion, we have made far apart the sleep groupings (wake and Random eye moment) [37,38], the subjects (bruxism and normal) and the combined signals (bruxism and normal), and the groups of the sleep (wake signal and REM signal) from ensemble machine learning classifier (EML) splits into classes.

The planned machine learning classifier is an amalgamation of ten machine learning classifiers in the Weka tool, including Sequential Minimal Optimization (SMO), Naive Bayesian (NB), Ad Boost (AB), Decision Tree (DT), Random Forest (RF), PART, which is similar in nature to CART algorithm which refers to classification and regression tree and PART has no full form as it builds a partial C4.5 decision tree in each iteration and makes the best leaf into a rule, Decision stump (DS), Logistic Regression (LR), Bayesian Net (BN) and multilayer perceptron (MP). There are two general disorders in an earlier analysis of sleep disorders. Primarily, it is shown that an ample number of applicable structures in the subjects give rise to a prominent computing load. Secondly, many approaches did not signify the classification of sleep. This analysis has resolved these problems. In future work, the main concentration is on some particular problems involving the estimation of power spectral densities, the application of new EML classification of distinctive approaches in the same area, and a comparative analysis performed among the EML classifier and earlier used class separators [39,40]. The signs and symptoms of bruxism consists of teeth crushing that may be sufficient for waking up your partner. The teeth due to crushing becomes distorted, weak and the enamel of the tooth is also lost and the inner coating of the teeth is exposed to air. The manuscript has been divided into four sections. The material and dataset have been considered in the first and foremost section. In the next section, the feature extraction approaches have been accomplished on the taken dataset. In the third segment, the ensemble and individual classification have been performed for the detection and analysis of the combined signal. The results and analysis have been considered in the last section, and then in Section 5, the conclusion has been illustrated.

## 2 Background & significance

Bruxism is a type of sleep disease in which persons clutch, masticate, and chore their teeth. It is described as an abnormal practice containing spontaneous recurring in functional clenching, crushing, or clasping of teeth [41]. If the subject crushes their teeth during the awake condition, it is known as awake bruxism; otherwise, if the subject crushes their teeth during their snooze time, it is known as snooze bruxism [42]. The indications of bruxism are crushing teeth, flattening, broken teeth, discomfort and compassion in teeth, jaw dysfunction, and facial ache, leading to sleep instabilities [43]. The research exposes 16.8% broad disparity, insufficient detection, and 21.9% of the candidate's intentional neural behavior action [44]. An entire number of 16 persons were utilized in this research. The authors advised that one channel is EMG and urge value is feasible for the diagnosis of bruxism disease. Here we planned a novel diagnosis scheme for bruxism utilizing the extraction of power spectral density on physical recordings of sleep including ECG (ECG1-ECG2), EMG (EMG1-EMG2), and EEG (C4-P4 and C4-A1) [45,46]. Ensemble approaches associate various basic models to form a more reliable and precise forecast mode. In the framework of bruxism diagnosis, the complication and inconsistency of signals from EEG, ECG and EMG recordings need an erudite technique that can efficiently solve distinct datasets designs. The selection of an ensemble approach such as random forest, gradient boosting or AdaBoost among the existing machine learning approach has various benefits that they are robust and can capture relationship among huge amount of data in the multiple signals. The algorithms that are used can decrease overfitting issue as compared to the specific models. The detection of bruxism data is imbalanced, where positive cases like bruxism instances are crucially less than the negatives. The ensemble approaches can handle this data properly. The algorithms used in the paper is a good combination as they can identify the features which are crucial for the bruxism detection. It can also handle the non-linear relationships like sensitivity and the importance of feature in a more effective manner that are complex for signal analysis. The performance to show the comparison is of good importance. The scalability and interpretation regarding the efficacy and the ability to interpret outcomes, particularly in clinical aspect where interpretation is important [[47], [80]].

## 3 Materials & methods

The data set used here is having the cyclic alternating pattern which is a periodic event of EEG at the time of NREM sleep, and distorted sleep patterns of CAP that are combined with sleep disorders. The CAP sleep data is a gathering of 108 polysomnographic recordings that has been collected from Ospedale Maggiore of Parma, Italy. Each sample consists of the recording of EEG signals together with tibial EMG signal and ECG signals. The two types of datasets have been taken into consideration one is bruxism, and the other one is normal. The data is taken from the Physio net website, which provides free access to normal and bruxism-affected persons noted data [48]. The indications of this data involve electroencephalography, electrocardiogram, electromyogram and electrooculogram and breathing [49]. The dataset used is taken from the open access that is physionet.org as the real time dataset is difficult to collect in case of bruxism patients. The approach planned used for the diagnosis of bruxism are collection of data, preprocessing of all the physiological signals, estimation of the normalized values and validation of the system with standard system. The normal that is healthy and bruxism patients were classified using different form of classifiers as shown in the figure below.

**Fig. 1:**
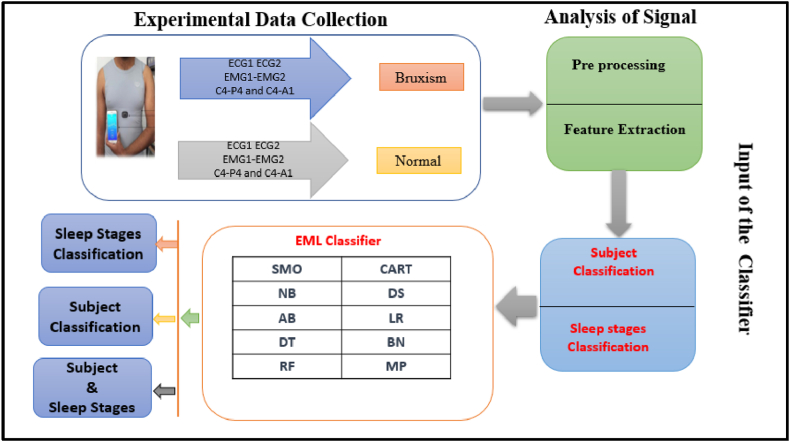
Organizational structure of the proposed work.

The real-time nursing of the chronicled dataset includes two state EOG, six channels of EEG signal, and two right and left leg EMG channels; subsequently, lower EMG and nasal breathing thermistors have been used to estimate the nature of respiration and the ECG signal [24]. The frequency of the sample values of the dataset taken is 200 Hz. All the considered signals have been taken to diagnose bruxism disorder in this research work. The recording of the single sign is 60 s. The entire networks have two kinds of sleep classes including wake and REM. A total of 56,160 sections are consumed in the proposed work. The dataset that has been taken has been clearly illustrated in Table 1. The approaches that apply to the diagnosis of bruxism have been described. The dataset has been taken from the sleep database to detect the disorder of sleep, bruxism. The next step, which is followed, is that the physiological signals have been extracted from the considered channel. Here the networks of the signals are pre-processed and the channel feature extraction has been performed. Preceding further, the channels are normalized before applying EML classifier. The process is described in the following Fig. 1. Two types of classifications have been performed one is subjects, and the other one is sleeping stages. The subjects are classified into bruxism and normal, and the other one is sleeping stages which are classified into the wake and random eye moment, and one is the collective classification (both persons and stages of sleep) were accomplished by applying the new EML (Ensemble machine learning) classifier. The organizational structure of the planned work is illustrated in Fig. 1 below.

**Table 1:** Data of the proposed scheme.

Description of the physical signal	Network of the physical signal	Number of persons (n)			Number of Sections (n)			Time of the signal
Signal	Signal	Man	Woman	Total	Bruxism	Normal	Overall	(s)
Electrocardiogram	Electrocardiogram1- Electrocardiogram	7	4	11	150	96	246	14,641
Electromyogram	Electromyogram-1 Electromyogram2	7	4	11	150	96	246	14,641
Electroencephalogram	C4-P4	5	5	10	141	85	226	13,441
Electroencephalogram	C4-A1	5	5	10	141	86	227	13,441
Mean		6	3.6	9.6	144.6	89.6	234.2	14,041
± SD		1.354	0.578	1.932	5.196	6.351	11.547	692.821

### 3.1 Power spectral density

The power spectral density using the Welch approach has been performed that was manufactured in 1967 [50]. The time series changes the section of data estimating the changeable periodogram of each section and calculating the average of the power spectral density [51]. The average changeable periodogram needs to decrease the variance parameter [52]. In future, it calculates the relative to an individual periodogram to cumulative data. PSD gives signal power with respect to the frequency spectrum. The number of frequency sections is there to assign the power spectral that is known as the number of fast Fourier transforms [53,54]. The Welch approaches are illustrated by the following equation below from (1), (2), (3)

(1) $$U=1/L\sum \_(n=0\hat{)}\left(L-1\right)▒\{w\_hm\;\left(n\right)\hat{\}}2$$

(2) $$P\_w\;\left(f\right)=1/LU\sum \_(n=0\hat{)}\left(L-1\right)▒\{w\_hm\;\left(n\right)x\left(n+iD\right)\;\hat{e}\left(-j2\pi fn\right)\;\hat{\}}2$$

(3) $$P_{w}\left(f\right)=\gamma \sum_{n=0}^{L-1}\left({\left\{X_{a}^{n}\right\}}^{2}+{\left\{X_{b}^{n}\right\}}^{2}\right)$$

here U is abbreviated as the parameter to reimburse for the loss of the signal, L and D are the datasets of the section whm(n) tends to the window of the hamming, γ is the constant, $X_{a}^{n}{\text{and}\;X}_{b}^{n}$ are the real and the imaginary part of the nth section, and Pw(f) is the power of the welch approach.

### 3.2 Ensemble machine learning (EML) classifier

There are various machine learning classifiers available to detect sleep disorders by review of different numbers of papers. The study's main aim is the assortment of the appropriate categorizer for competent diagnosis of sleep disorder. Though, the pursuit of machine learning classifiers changes from one disorder to another. Therefore, we have selected the combined machine learning classifiers to develop a new EML classifier. In this research, the ten classifiers considered for detecting SMO, NB, AB, DT, RF, PART, LR, BN and MP are available in the Weka toolbox, a systematic open-source software [55,56]. The machine learning classifiers have been combined for the analysis of the output by the majority elective in which we have taken out the outcomes of the ten classifiers which are taken for the majority of the outcomes. The example can explain it, if nine classifiers have been taken and the outcome is bruxism and one classifier outcome is healthy, then bruxism is the majority [57]. Therefore, the last outcome would be a sleeping disorder.

The EML categorizer enhanced the outcomes and decreased the fault of the scheme. In the research work, different categorizers basis on the amalgamation of numerous ML analyses were planned for the different anther complications. For example, Chen et al. [58] intended an amalgamation of machine learning analysis to detect the intellectual radio network. Rawa et al. [59] intend an EML ensemble ML scenario using an artificial neural network and Naïve Bayes to detect instructive pursuance in information removal. Miskovic [60] advised an ensemble model for classifying the choice provision. Lobbezoo et al. [5] proposes that bruxism is a monotonous oral event followed by crushing of teeth and specifically sleep bruxism or awake bruxism. Additionally, scaling scheme was planned to find out the probability of some evaluation of bruxism which produces the right output. The analysis produces the requirement for a good consciousness that has some goals as follows: (i) to clear the derivation and to form the different definitions for sleep and awake bruxism (ii) to find that bruxism is a syndrome or a nature which is bad for medical situations (iii) to re-evaluate the 2013 scheme (iv) to form a research goal. It was summarized that sleep as well as awake bruxism are oral muscle events that happens in the duration of frequent events subsequently.

### 3.3 Evaluation of the proposed system

In this planned research, a new 30-fold opposite validation model of the EML analyzer to distinguish the sleep disorder and normal persons. It is estimated with 235 ± 11.548 recordings of the considered channels, which involves 144.6 ± 5.197 sleep disorder and 89.6 ± 6.351 normal recordings, with C4-P4, and C4-A1 channels, which involves 144.5 ± 5.196 bruxism and 89.5 ± 6.350 healthy recordings, time duration of 14.040 ± 692.820 s. The estimation of the classification is applicable in the considered channels of the physical signals. Earlier, the channels are taken individually. Therefore, in the planned literature, the four channel to invent the efficient sleep disorder result is detecting bruxism. It has enclosed an entire signal [61].

The validity of a diagnostic test is determined as the ability of a test to tell who have the disease from who do not. For this purpose, two components are calculated: sensitivity and specificity. Sensitivity is the ability to correctly identify those who have the disease, while specificity is the ability to correctly identify those who do not have the disease. In order to calculate sensitivity and specificity, it is required that patients be identified by another test which provides a more permanent result, is often more sophisticated, more invasive, and more expensive, named gold standard. In our study, the polysomnography (PSG) was considered the gold standard for SB assessment.

The evaluation of the validity of a diagnostic test is usually performed on selected contexts as well, with two equally-numbered groups of patients—one with the disease, one without it—as this is an efficient way of describing sensitivity and specificity. Having that in mind, we selected a control group with the same number of patients as the group of patients with the disease for the sample, then we calculated the sensitivity and the specificity of each tested diagnostic criteria. We know that sensitivity and specificity are characteristics of the diagnostic test, although the predictive value is also influenced by the prevalence. That is why when we want to evaluate the discriminatory capacity of a particular diagnostic test, we calculate sensitivity and specificity, even though the predictive values are clinically more useful. In our study, besides the main measures to determine accuracy, we chose to present additional analyses reporting predictive values of each test, which were calculated based on the prevalence of our sample and not from the literature, despite the limitations. However, our conclusion was mainly based on sensitivity and specificity.

The concluding final pursuance of the classical by applying distinct renowned limits which are illustrated in the following equations: (4), (5), (6), (7), (8)

(4) Sensitivity=(TP/(TP+FN))

(5) Specificity=(TN/(TN+FP))

(6) Accuracy=((TP+TN)/(TP+TN+FP+FN))

(7) $$\text{MCC}=\left(\left(\left(\text{TPXTN}\right)‐\left(\text{FPXFN}\right)\right)/\sqrt{\left(\left(\text{TP}+\text{FP}\right)\left(\text{TP}+\text{FN}\right)\left(\text{TN}+\text{FP}\right)\left(\text{TN}+\text{FN}\right)\;\right)}\right)$$

(8) $$F‐\text{Measure = }\left(\left(2*\;\text{Precision}*\text{Recall}\right)\right)/\left(\text{Precision}+\text{Recall}\right)$$

where TP is abbreviated as the true positives, FP is abbreviated as the false positives, TN is abbreviated as the true negatives, and FN is the false negatives.

## 4 Results

### 4.1 Examination of the psychological signals

The 10/20 normal sleep recording scheme accounts for the physiological channels of sleep. For this, in the first step, each channel is extracted and filtered with a 201th-order low-pass infinite impulse response filter with an individual frequency of 25 Hz. The filtration is followed by the power spectral density classification of every physiological signal, then after values are normalized by classifying into subjects, sleep, sleep stages and also associate by applying EML classifier. The outcomes are presented by utilizing a new EML classifier. In earlier studies, the detection of bruxism is a sleep disorder, and stages are diagnosed by applying a decision tree classifier with a precision of 81.25%. Data mining is an approach used to fetch information from large datasets by analyzing data utilizing various algorithms that can assist in fetching crucial information from large datasets. Data mining stretches many algorithms to a bunch of datasets. It forms the data important and informative to attain the aim by relying on individual approaches to investigate and analyze the dataset to gather the best information. In the healthcare sector, data mining is one of the best approaches, which can create vast amounts of data about patients, detects, disorders and many more. In the healthcare sector, the eminence of serving is a general problem that medical organizations grieve. The eminence of detection of the disorder precisely and giving the correct suggestion, the poorer detection leads to dangerous outcomes that the clinical organization handles. The dataset considered for this research is in ARFF format, that is, attribute-relation file format, which has been gathered from physio-net.org, an open-access platform. The attributes here describe the binary class attribute. The tool used for analysis is Weka tool, an open-access toolbox [62,63].

Weka (Waikato environment for knowledge analysis) is a conventional set of machine learning programming based on Java script and created by the University of Waikato, New Zealand. The Weka set comprises a group of illustration algorithms and devices to analyze the dataset [64]. The nature of the dataset is in ARFF format, which is to be read by the tool in Weka. The Weka surveyor will apply these logically if it does not observe a given text as an ARFF recording. The pre-processing is applied to the ARFF dataset for denoising, and feature extraction is performed by applying different filters. The useful channels of the signals are extracted to classify the dataset [57]. There are many research investigations that has been conducted in the area of bruxism but what has been described in this manuscript is that we had used Weka tool for classification which is the easiest way of classification. The algorithm here used are the most efficient for the dataset taken and gives very good accuracy as compared to the existing literature. The strength of the algorithm is that it gives the results describing the effect of all the physiological signals on sleep whereas the existing literature gives the analysis of two signals or any one signal. The weakness of the algorithm is that it does not able to define any prosthetic structure or any hardware to reduce the effect of bruxism.

### 4.2 Data preprocessing

The next phase in data mining is pre-processing of the dataset, in which the transform is applied to propose the data mining algorithm. Then the selection of attributes is performed, then the missing data is handled to eliminate the outlier with five attributes selected to pre-process the dataset with 244 instances with 0 missing values. The following step is to mine the data with ten combined ensemble machine learning algorithms to investigate the difference in the various important parameters like accuracy, F1 score, recall, MCC and many more to see the efficiency of each combined algorithm. Ten-fold and Twenty-fold cross-validation is efficient enough in estimating the pursuance and computation time of the classifier. The primary phase is to find the number of instances of the sleep disorder data by applying all ten algorithms. Then it estimates the classification precision and cost analysis using a confusion matrix consisting of evidence about definite and predicted classification [57]. There are certain ordinary relations well-defined for the medium as follows: True Positive if the outcome of prediction is p the real one and in addition to that, it is known as True Positive (TP), False Positive (FP) if a real parameter is n and the unreal parameter is termed as false positive. The precision is determined depending on the above parameters [64].

**Fig. 2:**
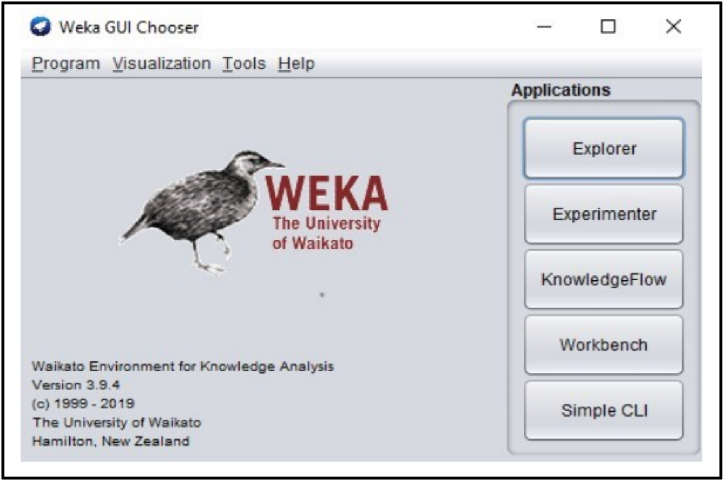
Weka GUI chooser.

**Fig. 3:**
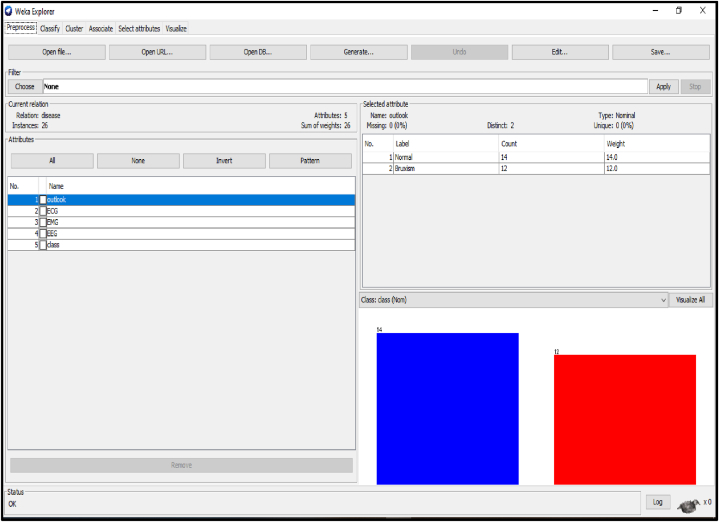
Graph showing the pre-processing of the collected bio-signals.

**Fig. 4:**
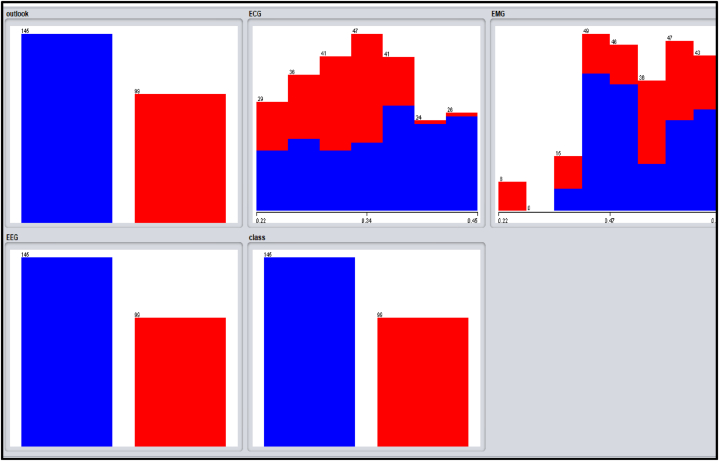
Graphical representation of all the physiological signals taken.

**Fig. 5:**
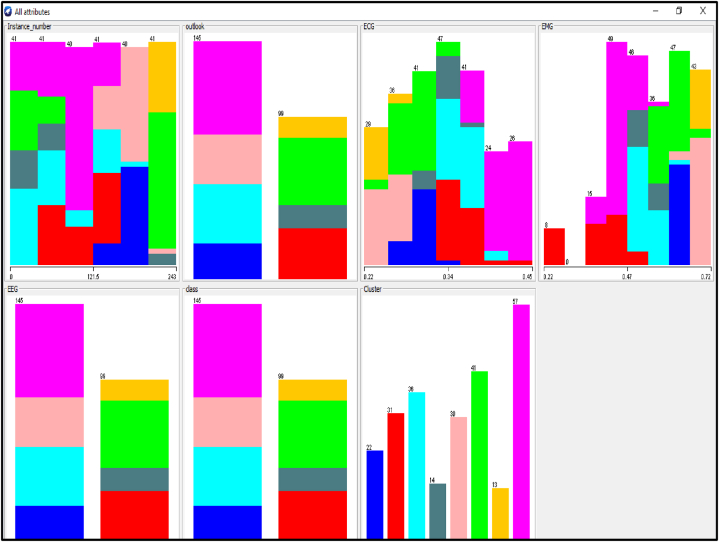
Graphical representation of the cluster analysis taken to the number of instances and the physical signals.

Several distinctive functions are given for data pre-processing, visualization of different attributes, classification, regression, clustering, feature selection and reduction. When we begin to work on the GUI server of Weka, it allows the user to select from the five kinds of submissions illustrated in Fig. 2: explorer, experimenter, knowledge flow, workbench and simple CLI. The research work done in this manuscript has been performed by selecting explorer application, and the following process is completed. Fig. 3 depicts the initial stage in the software in which the signals go through the pre-processing process, in which different filters are applied to remove noise in the signal. The red and blue graph in the figure shows the number of healthy and bruxism samples shown. Fig. 4 illustrates the graphical representation of all the physiological signals taken: ECG, EEG and EMG with the number of instances taken. In the figure it gives all the parameters taken for analysis which can be visualized through the graph. Fig. 5 depicts the graphical representation of the statistical parameters taken to the number of instances and the physical signals and it also shows the cluster analysis of the signals taken. The Weka GUI chooser has been illustrated in the following Fig. 2 below.

### 4.3 Clustering

**Fig. 6:**
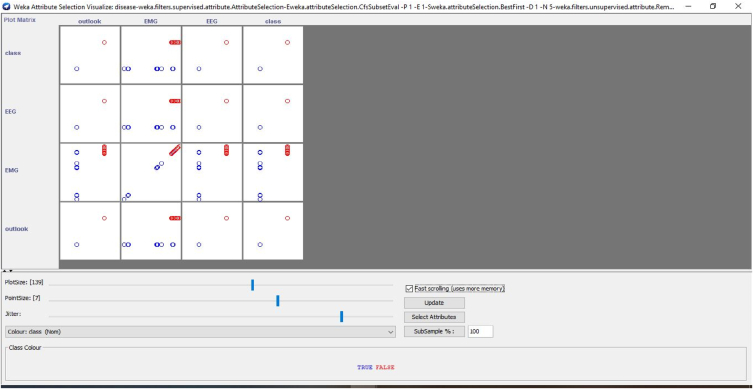
Graph showing the attributes and instances of the collected bio signals.

**Fig. 7:**
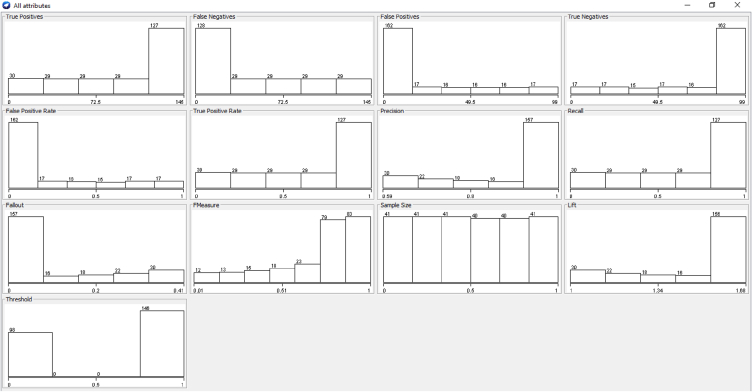
Graph showing the statistical parameters of the collected bio signals.

**Fig. 8:**
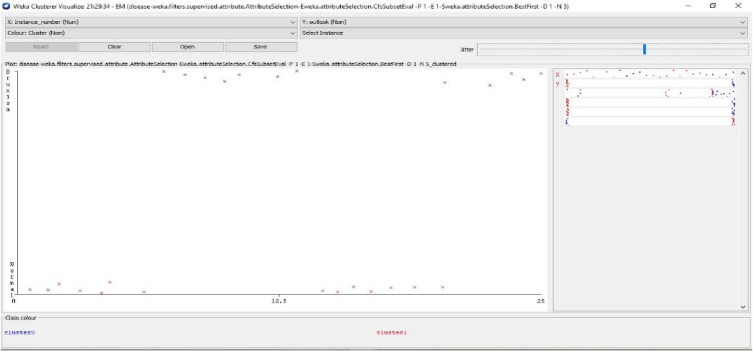
Graphical representation of class to cluster.

**Fig. 9:**
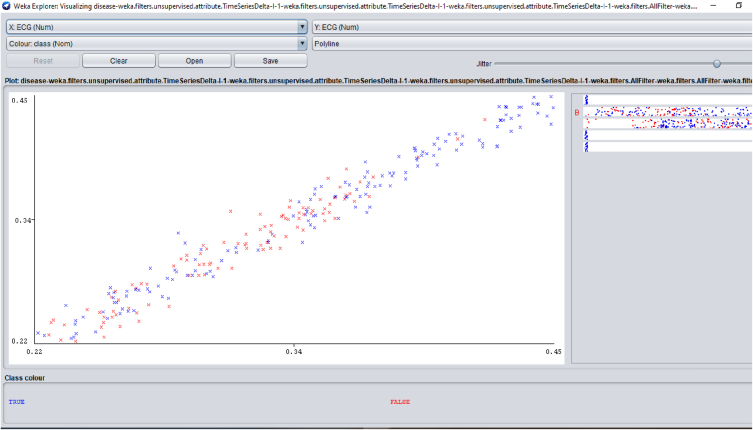
Classification value between bruxism and normal patients of the collected bio signals.

In the Weka tool, the clustering algorithm is a cluster of the same data instances. It assists various clustering algorithms including EM, filtered clutterer, hierarchical cluster, simple K-means clustering and many more. We may comprehend the algorithms entirely to activate the abilities of Weka fully. In the case of classification, Weka permits us to imagine the detected clusters explicitly. The clustering approach can be illustrated by giving the dataset. The data consists of three classes of 50 instances in each attribute [64,65]. The individual class denotes this type of group. Fig. 6 illustrates the graphical representation of the different attributes and the number of instances for the combined physiological signals that are EEG, ECG and EMG with the class. Fig. 7 describes the statistical parameters of the collected bio signals. The classification is done into two classes one is normal patients, and other one is bruxism. The graphical representation of class to cluster. Fig. 8 gives the classification Value between Bruxism and Normal Patients of the collected bio signals. Fig. 9 illustrating cluster analysis of EMG1-EMG2, ECG1, ECG2, C4-P4, and C4-A1 channels.

**Fig. 10:**
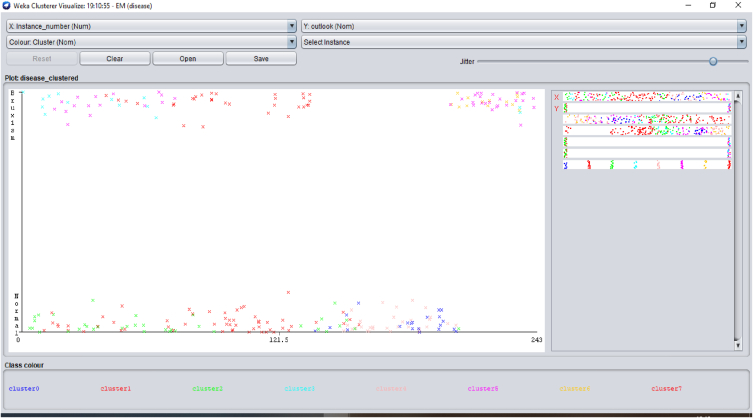
Cluster analysis of EMG1-EMG2, ECG1, ECG2, C4-P4, and C4-A1 channels.

### 4.4 Cost benefit analysis

**Fig. 11:**
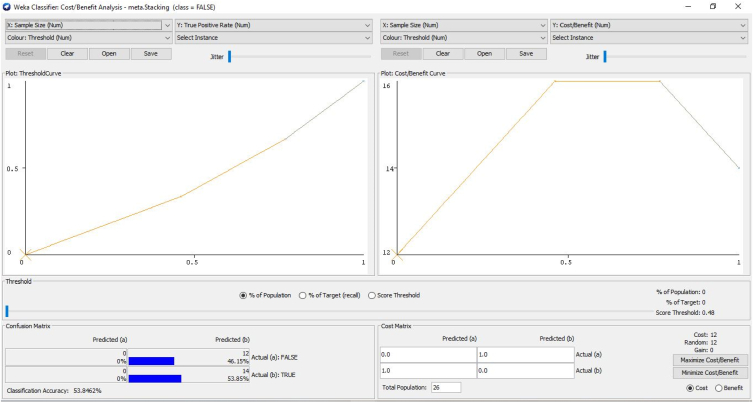
Cost analysis false combined classification for the present class of EMG1-EMG2, ECG1, ECG2, C4-P4, and C4-A1 channels.

**Fig. 12:**
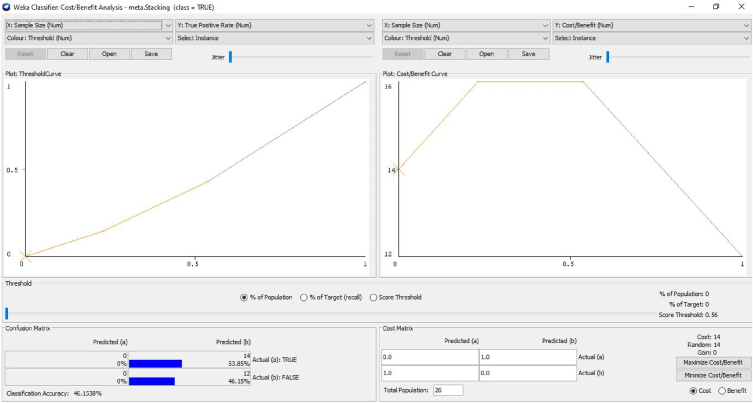
Cost benefit analysis true combined classification.

**Fig. 13:**
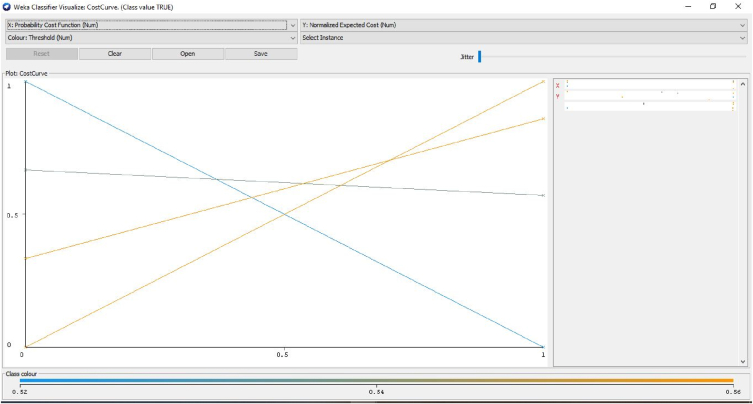
Representation between the probability cost function and the normalized expected cost.

The cost-benefit analysis values the span of bruxism from the patients to investigate the recording statistical parameters of wake level or with different dealing expressions. In addition to this, the investigation discovers the counts. It sums up all the optimum constraints related to advantages, then detects counts and decreases all the undesirable relations known as “Cost”. The alteration among these two values that are subjective to the proposed approach is suggested or not for the patients. The cost-benefit analysis could assist medical consultants in investigating the patient ratio with their clinical statistics and phases [66,67]. It also deals with the forecasting of tested optimum and pessimistic values. In the planned prototype, an upright forecast fraction is, as shown in Fig. 11 illustrates the cost Analysis False combined classification for the present class of EMG1-EMG2, ECG1, ECG2, C4-P4, and C4-A1 channels where the forecast for the optimum investigation is 53.85%. The undesirable investigation is 46.15% with the entire profit of 93% and 7.7% with the threshold. Fig. 10 gives the cluster analysis of all the physiological signals for all the considered channel of the dataset. Fig. 11 illustrates the cost-benefit Analysis of the true combined classification. Fig. 12 in describes the representation between the probability cost function and the normalized expected cost. Fig. 13 in gives the relation between the margin value and the cumulative value function of the channel.

**Fig. 14:**
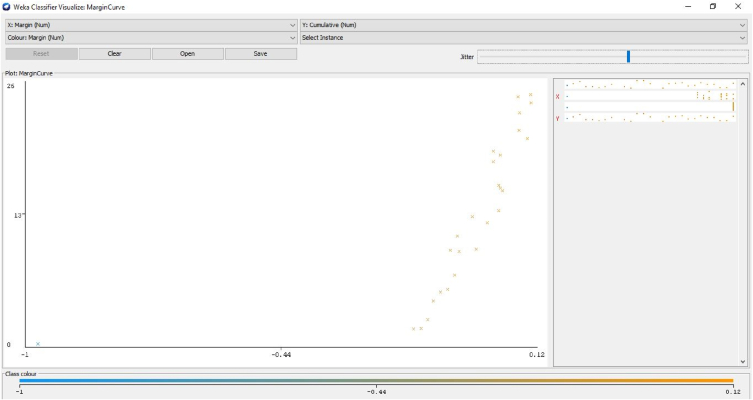
Relation between the margin value and the cumulative value function of the channel.

**Fig. 15:**
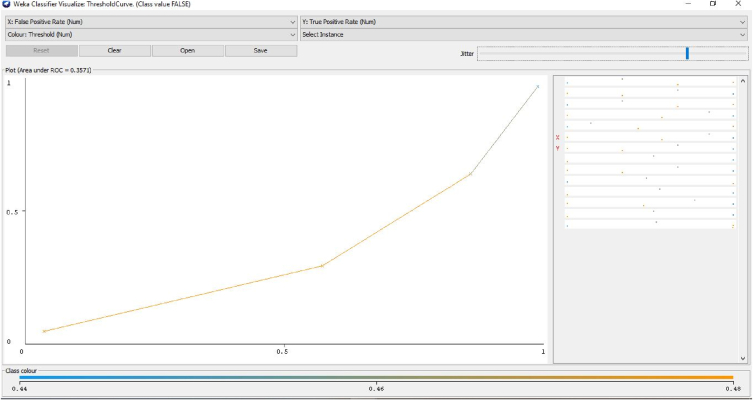
Threshold value between the true positive rate and false positive rate.

Fig. 14 depicts the relation between the margin value and the cumulative value function of the channel. Fig. 15 illustrates the threshold value between the true and false positive rates.

## 5 Discussion

### 5.1 Classification results using proposed novel EML classifier algorithms

This section elucidates the classification algorithms of all the ten algorithms considered for this research work. The SMO denotes the individual effective optimization algorithm applicable in the SVM algorithm, termed Sequential minimal optimization. The Algorithm 1 is as given below.

Algorithm 1 Sequential Minimal Optimization Classifier

**Table:** 

STEP:1 Import hyper parameters and data input
STEP:2 The linear classification of the above parameters will be calculated
STEP:3 Computing the constraints taken
STEP:4 Calculating the error and eta
STEP:5 Calculating the Lagrange multipliers
STEP:6 Calculating the threshold value

In statistics, Naïve Bayes is a simple probabilistic classifier that implements Bayes theorem with robust independent considerations between the constraints. The features are in the simplest Bayesian network models’ range but are associated with kernel density calculation and could attain a good precision value [68].

Algorithm 2 Naïve Bayes Classifier

**Table:** 

STEP:1 Import basic libraries
STEP:2 Import the dataset
STEP:3 Data pre-processing
STEP:4 Training the model
STEP:5 Testing and evaluation of the model
STEP:6 Visualizing the model

AdaBoost is an ensemble machine-learning algorithm for classification. It is a section of a cluster of ensemble approaches known as boosting that sums up novel machine learning models in a sequence where respective models' efforts to correct the forecast errors made by preceding models.

Algorithm 3 ADA Boost Classifier

**Table:** 

STEP:1 Assign equal weights to all observations. Initially assign same weights to each record in data
STEP:2 Classify random samples using stumps
STEP:3 Calculate total error
STEP:4 Calculate performance of the stump
STEP:5 Update weights
STEP:6 Update weights in iteration
STEP:7 Final Predictions

A J48 Decision Tree classifier augments the channels of the physiological signals. The process tree is designed to present the clustering process in the decision tree. The inner nodes of the tree show a test on the attributes, the tree branch gives the outcome of the test, the leaf node gives a mark of the class, and the node is the node's origin. The decision tree gives the outcome of the tree forecasts new dataset cases [69].

Algorithm 4 J48 Decision Tree Classifier

**Table:** 

STEP:1 INPUT D // Training data
STEP:2 OUTPUT T // Decision Tree
STEP:3 DTBUILD (*D) (T-Null)
STEP:4 T = Add arc to root node for each split predict and label
STEP:5 For each arc do
STEP:6 D = Database created by applying splitting predict to D
STEP:7 If stopping point reached for this path, then
STEP:8 T = Create leaf node and label with appropriate class
STEP:9 Else T’ = DTBUILD (D)
STEP:10 Add T′ to arc

While building a tree, J48 discards the missing value and permits classification by decision tree or formula produced by them. Random forest is an ensemble machine learning algorithm that can be applied for classification, regression and other tasks [70]. It implements by building an assembly of trees during training and giving rise to a predicted class. In Weka, to implement the RF algorithm select the algorithm from the cluster of trees.

Algorithm 5 Random Forest Classifier

**Table:** 

STEP:1 Select random samples from a given data or training set
STEP:2 This algorithm will construct a decision tree for every training data
STEP:3 Voting will take place by averaging the decision tree
STEP:4 Finally, select the most voted prediction outcome as the final prediction result.

CART refers to classification and regression tree, a predictive algorithm in machine learning, and it elucidates how the target constraints can be forecasted depending upon other values. It is a decision tree where each vertical is divided into a forecaster parameter, and each node has a forecast for the target value in the last.

Algorithm 6 Cart Classifier

**Table:** 

STEP:1 Select root nodes (S) based on Gini Index and highest information gain
STEP:2 Select node base on lowest Gini index or highest I.G.
STEP:3 Then splits set S to produce the subsets of data
STEP:4 An algorithm continuous to recur on each subset and make sure that attributes are fresh and creates the decision tree.

A decision stump is a part of a decision tree algorithm and of a similar nature. This only calculates the discrete attributes, meaning that the tree consists of one node and attributes are in number, and the tree can be made more complicated.

Algorithm 7 Decision Stump Classifier

**Table:** 

STEP:1 Start the problem with one main decision
STEP:2 Add chance and decision nodes
STEP:3 Expand until you reach end points
STEP:4 Calculate tree values
STEP:5 Evaluate outcomes

An LR algorithm is a binary classification for the input values considered for distribution. The last point need not be true as it can still attain optimum outcomes if the Gaussian data is not optimum. The algorithm trains the coefficient for individual input parameters, which is linearly associated with the regression function and changed using a logistic function. Logistic regression is a fast and straightforward approach and is very efficient on critical problems. The algorithm is described below.

Algorithm 8 Linear Regression Classifier

**Table:** 

STEP:1 Initialize the parameters
STEP:2 Predict the value of a dependent variable by given an independent variable
STEP:3 Calculate the error in prediction for all data points
STEP:4 Calculate partial derivative with respect to initial and final values
STEP:5 Calculate the cost for each number and add them
STEP:6 Update the initial and final values.

A Bayesian network model refers to an acyclic-directed graph. It is defined by applying one conditional probability distribution for individual constraints in the model, whose distribution is given dependent on its parents in the graph.

Algorithm 9 Bayesian Net Classifier

**Table:** 

STEP:1 Identify the main variable in the problem to solve. Each variable corresponds to a node of the network. It is important to choose the number states for each variable, for instance there are usually two states (true or false)
STEP:2 Define structure of the network that is the casual relationships between all the variables (nodes)
STEP:3 Define the probability rules governing the relationships between the variables

Multilayer Perceptron is an addition to feed-forward neural network. It contains three layers: the input layer, the output layer and the hidden layer. The input layer receives the input signal to be processed.

Algorithm 10 Multilayer Perceptron Classifier

**Table:** 

STEP:1 Starting with the input layer, propagate data forward to the output layer. This step is the forward propagation.
STEP:2 Based on the output, calculate the error (the difference between the predicted and known outcome). The error needs to be minimized
STEP:3 Backpropagate the error

**Fig. 16:**
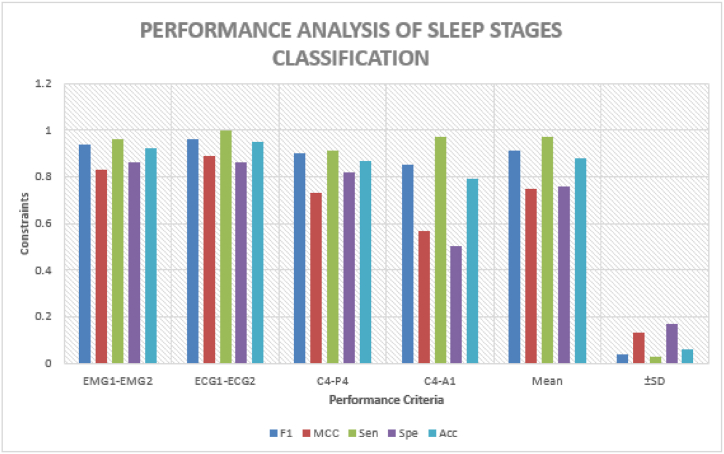
Comparison between all channels such as EMG1-EMG2, ECG1, ECG2, C4-P4, and C4-A1 channels of the sleep stages classification.

**Fig. 17:**
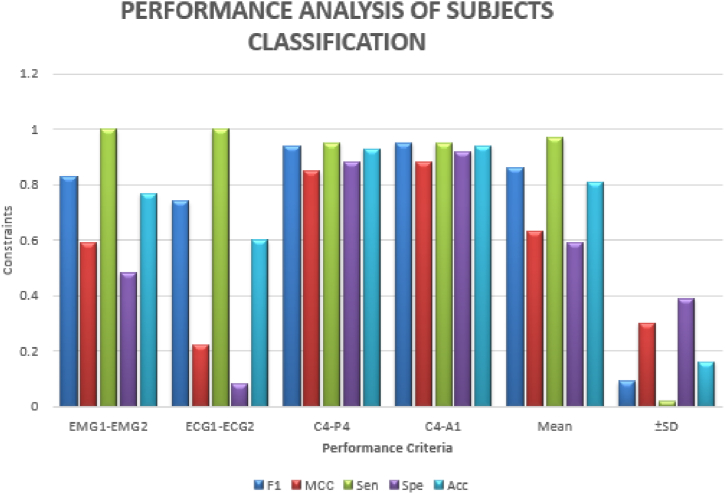
Comparison between all channels such as EMG1-EMG2, ECG1, ECG2, C4-P4, and C4-A1 channels of the subject's classification.

**Fig. 18:**
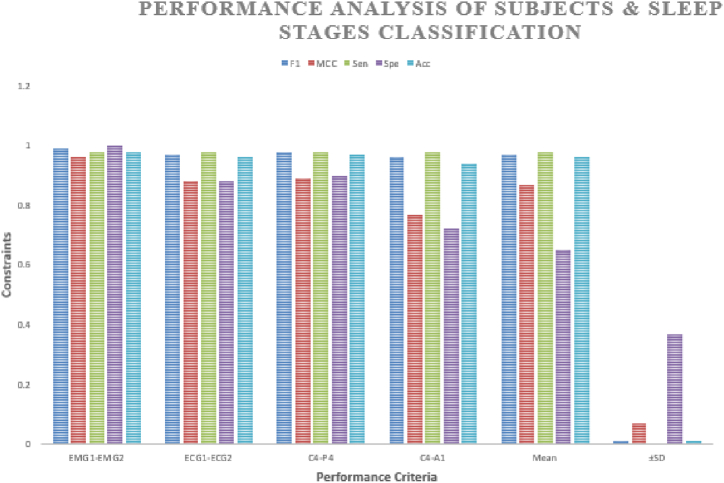
Performance analysis for the combination of subjects (bruxism and healthy) and sleep stages (w and REM) classification using the novel 20-fold HML classifier.

**Fig. 19:**
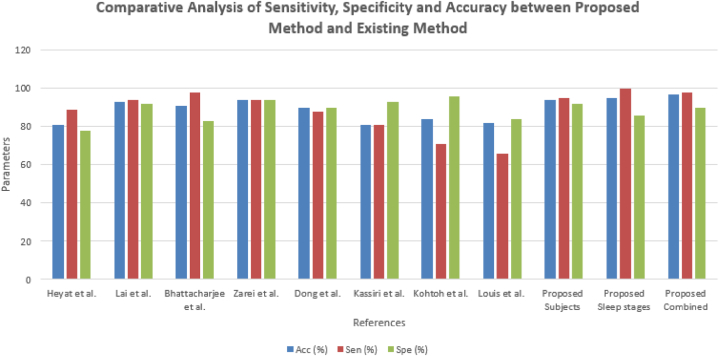
Comparative analysis of sensitivity, specificity and accuracy between proposed method and existing method.

**Fig. 20:**
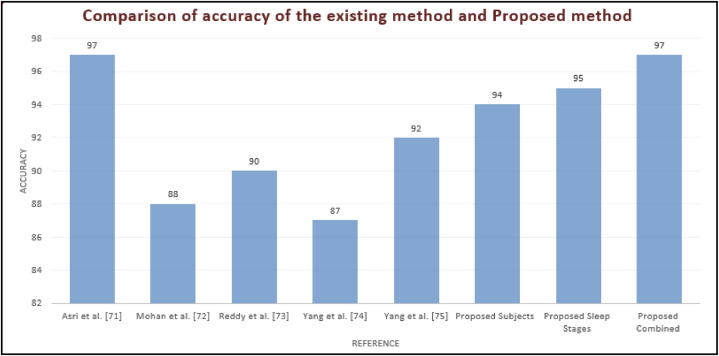
Comparison of accuracy of the existing method and proposed method.

The pursuit for classifying subjects which has been illustrated in Table 2. Earlier, the detection of bruxism sleep disorder and the stages of sleep by employing the DT classifier with a precision of 81.25% [43]. La et al. [34] illustrated the cardiac and muscle channels utilized for diagnosing sleep disorders by employing the power spectral density analysis approach. The 489 minimum datasets for the two channel and classification of the subjects and sleep stages are applicable using the ensemble ML approach. The planned scheme, that is, we have used the C4-A1 channel, has the finest pursuance in the persons (bruxism and healthy) organization which is the basis of sensitivity, specificity and precision of 95%, 92%, and 94%, subsequently. The pursuance of the stages of the snooze organization is illustrated in Table 3. Earlier, the unverified training building and concealed Markov model has been applicable for diagnosing stages of sleep [71]. Bo et al. [72] applied a multimodal sensor scheme estimating hand speed, cardiac signal and Actiwatch for detecting the stages of sleep including REM and NREM. Bajaz et al. [73] established an automatic method for detecting stages of sleep by applying time-frequency images of the EEG signals. Matsuura et al. [74] investigated that the cardiac degree dimension is assisting and relaxed, which is very helpful in stages sleep monitoring. The proposed scheme has estimated the four stages and the entire five stages precision which is 66% of the scheme. The planned scheme, the ECG1-ECG2 channel, has the maximum pursuance in the stages of sleep (w and REM) classification basis on sensitivity, specificity and precision that is 100, 86 and 95 % subsequently. Fig. 16 compares all channels of the sleep stages classification, such as EMG1-EMG2, ECG1, ECG2, C4-P4, and C4-A1 channels. Fig. 17 compares all channels of the subject's classification, such as EMG1-EMG2, ECG1, ECG2, C4-P4, and C4-A1 channels. Fig. 18 gives the performance analysis for the combination of subjects (bruxism and healthy) and sleep stages (w and REM) classification using the novel 20-fold HML classifier. Fig. 19 illustrates the comparative Analysis of Sensitivity, Specificity and Accuracy between the proposed and existing methods. Fig. 20 compares the existing and proposed methods' accuracy.

**Table 2:** Statistical comparative analysis among machine learning classifier.

Classifier	Accuracy	Recall	F-measure	Execution time	Kappa Statistic	Mean absolute error	MCC
SMO	97.16	0.92	0.62	0.80	0.76	0.79	0.86
NB	98.43	0.93	0.71	0.88	0.67	0.65	0.75
AB	94.32	0.91	0.78	0.89	0.69	0.63	0.70
DT	95.43	0.90	0.81	0.85	0.98	0.45	0.98
RF	96.17	0.89	0.83	0.83	0.84	0.63	0.73
CART	98.76	0.87	0.67	0.78	0.72	0.72	0.71
DS	99.03	0.93	0.89	0.71	0.63	0.81	0.90
LR	99.36	0.95	0.96	0.72	0.74	0.71	0.54
BN	98.16	0.96	0.94	0.74	0.96	0.64	0.43
MP	98.84	0.99	0.93	0.95	0.77	0.98	0.38

**Table 3:** Pursuance of the sleep stages (w and REM) classification using the novel 20-fold cross-validation model of the HML classifier.

Description of the Channel	Positive True	Negative True	True Positive	True Negative	False Positive	False Negative	Frequency1	MCC	Sensitivity	Specificity	Accuracy
Electromyogram1- Electromyogram2	55	28	51	26	5	2	0.95	0.84	0.97	0.87	0.93
Electrocardiogram 1- Electrocardiogram 2	57	26	53	26	6	0	0.97	0.90	2	0.87	0.96
C4-P4	48	28	43	24	6	5	0.10	0.74	0.92	0.83	0.88
C4-A1	60	16	46	15	15	2	0.86	0.58	0.98	0.6	0.80
**Mean**	55	23.6	47.26	21.76	6.76	1.76	0.92	0.76	0.98	0.77	0.89
**±SD**	5.10	5.75	4.57	5.26	4.85	1.71	0.05	0.14	0.04	0.18	0.07

### 5.2 Comparison of the proposed approach with the existing state of art techniques in bruxism

The pursuance of the person (normal and sleep disorder) organization of the EEG (C4-A1 network) sign concerning parameters of sensitivity, specificity, and precision were 95%, 92% and 94%. The pursuance of the stages of sleep (wake and Random eye moment) organization on the ECG (ECG1-ECG2 channel) of the signal concerning parameters sensitivity, specificity, and precision were found to be 100%, 86% and 95%, respectively. In addition to the pursuance of the associated subjects and stages of the sleep classification concerning subjects on the EEG (C4-P4 channel), signals relating to sensitivity, specificity and accuracy were subsequently subjected to 98%, 90% and 97%. The C4-P4 network of the associated classification has good pursuance than the others [75]. Earlier, sleep disorder diagnosing approaches were imperfect compared to the disorder of sleep and stages of sleep detection approaches. Bruxism-affected persons are standard by different detection approaches, including surveys, medical investigations and equipment. The surveys included the past patient of teeth flexibility and teeth accessory, muscle ache, the sensitivity of the tooth and crushing muscle uneasiness, tiredness or pain [76]. Medically, Ekfelt et al. [77] diagnosed a sleep disorder affected person by the internal mouth and external mouth investigation. Takeuchi et al. [78] advised that bruxism is more precisely restrained by the masticatory muscle EMG recordings and polysomnography.

The comparative analysis has been performed with earlier selected sleep disorders and stages of sleep approaches concerning authors, publications, subject diagnosis, sign, classifier, sen, spec and precision, as discussed. Table 2 gives the statistical comparative analysis among machine learning classifiers. Table 3 pursues the sleep stages (w and REM) classification using the novel 20-fold cross-validation model of the HML classifier. Table 4 performs the subjects (bruxism and healthy) classification using the novel 20-fold cross-validation model of the EML classifier.

**Table 4:** Performance of the subjects (bruxism and healthy) classification using the novel 20-fold cross-validation model of the EML classifier.

Name of the Channel	PT	NT	TP	TN	FP	FN	F1	MCC	Sensitivity	Specificity	Accuracy
Electromyogram1- Electromyogram 2	65	18	47	18	19	1	0.84	0.60	2	0.49	0.78
Electrocardiogram1-Electrocardiogram2	79	4	48	4	33	1	0.75	0.23	2	0.09	0.61
C4-P4	49	27	46	25	3	3	0.95	0.86	0.96	0.89	0.94
C4-A1	48	28	47	26	3	3	0.96	0.89	0.96	0.93	0.95
Mean	59.5	18.3	45.6	17.3	13.8	2	0.87	0.64	0.98	0.60	0.82
±SD	14.8	11.2	0.58	10.2	14.2	1.16	0.10	0.31	0.03	0.40	0.17

The dataset from the disorders of sleep and stages of sleep diagnoses approaches involving EEG, EMG and ECG signals. The various classifiers, including SMO, NB, AB, DT, RF, CART, DS, LR, BN and MP, are novel hybrid machine learning classifiers. The planned EML classifier attains acceptable pursuance in diagnosing bruxism. Table 1 illustrates the performance for the combination of subjects (bruxism and healthy) and sleep stages (w and REM) classification using the novel 20-fold cross-validation model of the HML classifier. Table 2 compares the proposed method with the existing methods. Table 3 illustrates the comparison of the proposed EML classifier with the existing ensemble classifiers. The planned sleep stages analysis has various advantages over the existing contribution. The first most advantage is that an ensemble machine learning algorithm can efficiently resolve the class imbalance issue in sleep stages. The second advantage of this proposed work is assumed to be one of the impacts of the age that is used to analyze the sleep patterns efficiently, that impacts enhancing the classification precision. The precision value of the proposed is good as compared to the existing approaches.

R and Python are useful in bruxism detection studies because they provide useful tools and libraries for signal processing, machine learning, and data analysis. Python is a popular choice for processing and analyzing huge datasets, including signals from EEG, ECG, and EMG recordings in bruxism investigations. Its numerous libraries, like as NumPy, Pandas, and SciPy, further enhance its usefulness. R offers a wide range of statistical analysis programmers, such as dplyr, tidyr, and stats, which facilitate thorough data exploration and manipulation. This is essential for comprehending the signal patterns linked to bruxism. By combining R and Python, researchers can take advantage of each language's advantages when studying bruxism detection. R's capabilities for statistical analysis and visualization are enhanced by Python's adaptability in managing data and modelling machine learning. With the use of libraries like SciPy and Scikit-learn, researchers may preprocess, filter, and extract characteristics from the various signals that are acquired from bruxism sensors.

R's statistical capabilities in conjunction with signal processing techniques make it easier to extract pertinent information from complex signal data, which helps in feature engineering and analysis. Python's TensorFlow, Scikit-learn, and Keras offer a stable environment for machine learning modelling. These libraries provide a range of algorithms that are appropriate for classification tasks, including as neural networks, ensemble approaches, and clustering algorithms that can be applied to the detection of bruxism. Data visualization is made easier by libraries like Matplotlib, Seaborn, and Plotly, which also help with the interpretation of findings and the production of visually appealing graphics. Additionally, R has machine learning tools such as random Forest, caret and xgboost, allowing researchers to use data related to bruxism to create predictive models and carry out categorization tasks. Table 5 gives the comparison of the proposed HML classifier with the existing hybrid classifier.

**Table 5:** Comparison of the proposed HML classifier with the existing hybrid classifiers.

Reference	Year	Disease	Acc (%)
Asri et al.	2021	Cancer	97
Mohan et al.	2020	Heart	88
Reddy et al.	2019	Heart	90
Yang et al.	2018	Schizophrenia	87
Yang et al.	2017	Cushing	92
**Proposed**		**Bruxism**	**94**
			**95**
			**97**

## 6 Application and limitation of the proposed system

The proposed system illustrates an application to diagnose bruxism disease and sleep phases using all the considered channels. This research gives an additional efficient and precise diagnosis scheme of bruxism and snooze phases for clinical applications. The most crucial implementation of the current investigation is to detect persons with a psychological healthiness situation quickly and precisely [79]. This scheme also assists in detecting the disorder in the duration of the sleep stage. The current work has few restrictions in that the presented data from the physical data were old and small persons for the estimation. In future, the research can be extended for massive amounts of real-time datasets with better precision. The presented physical signals, involving cardiac, brain, and muscle signals, covered only some sleep footage. Additionally, the snooze phases involve wake and Random eye moments. Further, we will apply distinct signs and stages of sleep to recognize sleep disorders.

These organizations are able to consistently access the bruxism signs and symptoms permitting them for primary diagnosis of teeth clenching. Early detection can appropriately conduct, stopping possible dental problems. Incessant nursing at home delivers a longitudinal opinion of bruxism designs, easing a healthier considerate of its occurrence, strength, and possible inductions over period. Home-based monitoring systems offer suitability, as affected role can experience nursing in their usual atmosphere without the restraints of scientific appointments. This often leads to improved obedience with the nursing routine. These schemes can be custom-made to separate requirements, providing personalized insights into bruxism behavior for specific patients. Such personalized data can assist healthcare professionals in designing tailored treatment plans. Incessant information gathering produces a prosperity of material that can be examined to originate visions into social designs related with bruxism. This data can help in filtering action plans. Real-time data show from home nursing systems can permit distant health care specialists to measure and interfere punctually, contributing to guide or adjust treatment plans as essential. Combined dataset from these schemes could donate to large-scale investigate studies, easing a deeper understanding of bruxism's prevalence, patterns, and potential associations with other health factors. Confirming the accuracy and consistency of home nursing strategies is vital. Erraticism in sensor correctness could influence dataset superiority. Safeguarding the correctness and consistency of home nursing plans is vital. Variability in sensor accuracy or patient compliance could impact data quality. Scheming schemes that are informal to use and understand for affected role is vital to ensure their sustained obedience and appointment with the nursing procedure.

## 7 Conclusion and future scope

Bruxism is the sleep syndrome in which people clinch, grind and gnash their teeth when in sound sleep. We have established a new EML classifier to diagnose bruxism for the planned invigilation. The outcomes illustrate that the EML classifier can efficiently distinctly subjects and the stages of sleep and these two combined (subjects and stages of sleep) with 94, 95 and 97% precision. In our information, this approach (EML classifier) is used for the primary duration to diagnose bruxism. We proposed the EML classifier's EEG (C4-P4 channel) signal that could apply in bruxism detection. This approach can obtain an efficient outcome with PSD with work application for the disorder of sleep and stages of sleep detection. The imminent study will fast diagnose sleep disorders with the highest precision.

The future research work in bruxism diagnosis gives a focus on the medical system and detection on the basis of diversified population like the different age groups as the sleeping patterns and duration of sleep required is different for different age-groups takes a promising approach in advancing and understanding the good management of this condition. Comparing the performance of diagnosis approaches over medical settings by medical experts or the standard medical tools would enhance the reliability of the methods used. Long-duration of analysis can offer the depth growth of bruxism its importance and the effect of several interventions. Monitoring patients over long-duration can disclose the patterns and distortions in the nature of bruxism. Analyzing bruxism over diverse patient populations assuming the attributes like age, gender, their background can enhance the changes in relevance, sternness and work getting factors. Strategies for diagnosis and treatment could be customized for various groups with the aid of this research. Strategies for diagnosis and treatment could be customized for various groups with the aid of this research. Improving patient care can be achieved by investigating the incorporation of remote monitoring technology into clinical practice. It is critical to evaluate if clinical settings' patients and healthcare professionals will accept such systems and if they are practical, useful, and acceptable. More thorough and reliable bruxism detection techniques may be available through research into the fusion of different signals (such as ECG, EMG, and wearables) or the integration of numerous sensor technologies (such as smartphone applications, dental devices, and wearables). Based on detection results in clinical settings, it is essential to investigate the efficacy of various therapeutic strategies. The effectiveness of behavioral interventions or customized treatment would be greatly impacted by an understanding of how early identification through monitoring translates into those results. Investigating bruxism's effects on health that go beyond tooth problems, such as how it affects stress levels, sleep quality, and general health outcomes, would offer a comprehensive picture of the condition's impacts.

## CRediT authorship contribution statement

**Pragati Tripathi:** Writing – review & editing, Writing – original draft, Methodology, Investigation, Formal analysis, Data curation, Conceptualization. **M.A. Ansari:** Visualization, Validation, Supervision, Methodology, Investigation, Formal analysis, Data curation, Conceptualization. **Tapan Kumar Gandhi:** Validation, Methodology, Investigation. **Faisal Albalwy:** Writing – review & editing, Methodology, Investigation, Funding acquisition, Formal analysis. **Rajat Mehrotra:** Methodology, Investigation. **Deepak Mishra:** Methodology, Investigation.

## Declaration of competing interest

The authors declare that they have no known competing financial interests or personal relationships that could have appeared to influence the work reported in this paper.
